# Facemask Usage Among People With Primary Ciliary Dyskinesia During the COVID-19 Pandemic: A Participatory Project

**DOI:** 10.3389/ijph.2021.1604277

**Published:** 2021-12-15

**Authors:** Eva S. L. Pedersen, Eugenie N. R. Collaud, Rebeca Mozun, Katie Dexter, Catherine Kruljac, Hansruedi Silberschmidt, Jane S. Lucas, COVID-PCD patient advisory group, Rindlisbacher Bernhard, Myrofora Goutaki, Claudia E. Kuehni

**Affiliations:** ^1^ Institute of Social and Preventive Medicine, University of Bern, Bern, Switzerland; ^2^ PCD support UK, London, United Kingdom; ^3^ PCD Australia Primary Ciliary Dyskinesia, Altona, VIC, Australia; ^4^ Verein Kartagener Syndrom und Primaere Ciliaere Dyskinesie Deutschland, Wetzikon, Switzerland; ^5^ Primary Ciliary Dyskinesia Centre, NIHR Biomedical Research Centre, University Hospital Southampton NHS Foundation Trust, Southampton, United Kingdom; ^6^ Faculty of Medicine, School of Clinical and Experimental Medicine, University of Southampton, Southampton, United Kingdom; ^7^ Division of Paediatric Respiratory Medicine and Allergology, Department of Paediatrics, Inselspital, Bern University Hospital, University of Bern, Bern, Switzerland

**Keywords:** facemask, mask, chronic disease, primary ciliary dyskinesia, rare disease, coronavirus

## Abstract

**Objectives:** Primary ciliary dyskinesia (PCD) is a rare genetic disease that causes recurrent respiratory infections. People with PCD may be at high risk of severe COVID-19 and protection against SARS-CoV-2 is therefore important. We studied facemask usage and problems reported in relation with their use among people with PCD.

**Methods:** We used data from COVID-PCD, an international observational cohort study. A questionnaire was e-mailed to participants in October 2020 that asked about facemask usage.

**Results:** In total, 282 participants from 27 countries were included (Median age 32 years; 63% female). In total, 252 (89%) wore facemasks everywhere in public, 13 (5%) wore facemasks in most places, and 17 (6%) did not wear facemasks in public. Half of the participants reported that it was uncomfortable to wear facemasks because of runny nose, cough, or difficulty breathing. Participants less often wore facemasks when there was no national requirement.

**Conclusion:** Most people with PCD wore facemasks despite frequent respiratory problems related to their use. Facemask usage was most frequent in countries with a national requirement emphasizing the importance of nationwide policies mandating facemasks.

## Introduction

Facemasks help prevent transmission of SARS-CoV-2 between people [1–3] and have become a key preventive measure in the COVID-19 pandemic [4–6]. Many governments around the world implemented mandatory facemask use in public spaces throughout 2020 [7]. Facemasks are especially important for people who are at high risk of severe COVID-19 such as elderly people or people with chronic diseases [8–11]. However, many people have reported discomfort resulting from the use of facemasks such as difficulty breathing, pain around the ears, headaches, or skin problems [12, 13]. High-risk populations such as people with a chronic respiratory disease may experience additional problems because of respiratory symptoms such as chronic cough and rhinitis [14, 15]. We found no original studies reporting on the frequency of facemask usage or on problems encountered by people with a chronic respiratory disease [16].

Primary ciliary dyskinesia (PCD) is a rare genetic multi-system disorder where dysfunctional cilia lead to impaired mucociliary clearance, laterality defects, and other health problems [17–21]. Most people with PCD have recurrent upper and lower airway disease resulting in chronic respiratory symptoms such as constantly runny nose and chronic cough with sputum production [17, 22]. Lung function in people with PCD is often reduced and can lead to oxygen requirement [23–26], and hearing impairment is common because of chronic otitis media [14, 20]. At the start of the pandemic, people with PCD or other chronic respiratory diseases were considered at high risk of a severe disease course and were recommended to ensure good shielding [27]. Shielding refers to all protective measures taken to avoid infection and includes mitigation of social contact, staying at home as much as possible, and wearing facemasks in public [28]. In May 2020, we set up an international participatory research study, COVID-PCD, to follow people with PCD during the COVID-19 pandemic [29]. By March 2021, 24 of 640 participants (3.8%) had reported a positive SARS-CoV-2 test but most reported mild or no symptoms [30]. However, they carefully shielded themselves by staying at home and avoiding public places. Another study in 27 children and adults with PCD also showed that all shielded carefully [31]. It is possible that people with PCD could be particularly burdened by facemasks due to chronic wet cough, constantly runny nose, hearing problems [14, 17]. We aimed to understand the usage of facemasks among people with PCD in an international context during the COVID-19 pandemic. Specifically, we assessed how often and which type of facemasks people with PCD wear, studied beliefs related to facemasks, described problems reported in relation with their use, and described characteristics of participants who did not wear a facemask in public.

## Methods

### Study Design and Inclusion Criteria

The data that we present here was obtained from a cross-sectional questionnaire on facemasks nested within the COVID-PCD study. The COVID-PCD is an international observational cohort study that uses anonymous online questionnaires to collect information directly from people with PCD during the COVID-19 pandemic (clinicaltrials.gov: NCT04602481). COVID-PCD is a participatory research project where people with PCD have an active role in all stages of research from the design of the study, its content, the piloting, and communication of results. Details about the study methods have been published [29, 30]. In short, the COVID-PCD study includes persons of any age from anywhere in the world with a confirmed or suspected diagnosis of PCD. The study is designed for three age groups; children below 14 years, adolescents between 14 and 17 years, and adults aged 18 years or more. For children, the questionnaires are addressed to the parents, but the child is encouraged to help complete the questionnaires. Adolescents and adults complete the questionnaires themselves. The study is available in English, German, Spanish, Italian, and French. Recruitment started on May 31, 2020. The Cantonal Ethics Committee of Bern approved the study (Study ID: 2020-00830). Informed consent to participate is provided online at the time of registration into the study. This article follows the STROBE reporting recommendations [32].

### Study Procedures

The COVID-PCD study is conducted online. Participants are invited by PCD support groups who contact and inform people living with PCD through social media and email networks and encourage them to take part. The website (www.covid19pcd.ispm.ch) includes detailed information about the study and allows participants to register anonymously and give consent to participate. After registration, participants receive an email with a link to the first questionnaire. Participants first complete a baseline questionnaire with questions on their disease, their usual symptoms, and SARS-CoV-2 infections experienced prior to joining the study. Thereafter they receive weekly follow-up questionnaires with questions on incident SARS-CoV-2 infections, current symptoms, social contact behaviour, and physical activity. Intermittently, questionnaires focus on special topics. This manuscript presents data from a special questionnaire focused on facemask usage which was sent to all participants on October 30th^,^ 2020. Collaborating PCD support groups were strongly involved in the development of the questionnaire. A draft of the special questionnaire on facemask usage was sent to seven representatives from the collaborating PCD support groups who suggested changes to the questions or proposed additional questions. The finalized questionnaire was then programmed in a Research Electronic Data Capture (REDCap) database (www.project-redcap.org) [33], and thereafter piloted by the PCD support group representatives before it was sent out to participants in the COVID-PCD study. The REDCap database is hosted by the Swiss medical registries and data linkage centre (SwissRDL) at the University of Bern, Switzerland, and complies with all legal requirements for data security and data protection [29]. Participants received up to two reminders if they did not respond to the first questionnaire.

### Information About Mask Use

The questionnaire asked whether participants used facemasks in public and if so, in which places, and whether they were exempt from wearing a face mask because of their disease (Supplementary Material S1). We also asked about problems people with PCD experienced when wearing facemasks and whether they needed to remove their mask because of these problems. We asked about their beliefs regarding effectiveness of facemasks, whether participants experienced communication difficulties, and whether costs of facemasks represented a financial burden to them. In the questionnaire for children, we asked if the child was too young to wear a facemask and if the parent said yes, the child was excluded from the analysis.

### Statistical Analyses

We described demographics of the participants, frequency of face mask use, and problems related to wearing a facemask using number and proportion for categorical variables and mean and standard deviation (SD) or median and interquartile range for continuous variables. We studied determinants of not wearing a mask in public by dividing participants into 3 categories, 1) always wears facemask, 2) does not wear facemask in one place, 3) does not wear facemask in two or more public places. These three categories were constructed from reported facemask usage in the following public places: grocery stores, clothes stores, restaurants, cinemas, hairdressers, physiotherapists, and post-/bank offices. We selected these places because facemasks were compulsory in those places in most countries at the time of the study (Table 2). We then compared characteristics of people always wearing face masks in public with people not wearing facemasks in one or more public places using Fisher’s exact test. We chose Fisher’s exact due to small sample size in certain categories [34]. We also tested whether people who came from countries with no national facemask requirement were less likely to wear facemasks in public compared to people coming from countries with a national facemask requirement (source data on national facemask requirements: https://masks4all.org/) [7]. Our dataset had few missing values (less than 1% in single variables) except for questions asking about who pays for masks (10% missing answers) and affordability of masks (15% missing answers). Records with missing values were excluded from the analyses. We used STATA version 15 for statistical analysis [35].

## Results

### Study Population

In total, 297 of the 572 people (53%) who participated in the COVID-PCD study in October 2020 returned the special questionnaire (completed between October 30 and November 12, 2020). We excluded 15 children because their parents reported them too young to wear a facemask regularly, and we finally included 282 people with PCD in this analysis. Median age was 32 years (age range: 3–85 years, interquartile range 17–48) (Table 1). Participants who completed the facemask questionnaire were older than those who did not complete it (median age of non-responders: 22 years (interquartile range 8–37) and more often came from European countries (Supplementary Material S2). Study participants came from 27 countries, with largest numbers from the UK (n = 62; 22%), Germany (n = 57; 20%), USA (n = 45; 16%), and Switzerland (n = 21; 7%). The majority of study participants (96%) came from countries where facemasks were mandatory in some or all public places (24 out of 27 countries at the time of the survey, Table 2). Twelve people came from countries with no national facemask requirement including Hong Kong, Norway, and Sweden (Table 2). Most participants used more than one type of facemask. Most common were reusable facemasks without exchangeable filters (n = 136, 48%) followed by certified single-use facemasks (n = 128, 45%), and single-use non-certified masks (n = 56, 20%), filtering face piece (FFP, FFP2, or FFP3) (n = 57, 20%), and fabric masks with exchangeable filters (n = 64, 23%) (Table 1). Few participants reported to have an exemption for wearing a facemask in public due to PCD; two participants (1%) had a personal facemask exemption prescribed by their physician, and 32 (11%) reported that in their region people with PCD were exempt from wearing a facemask because of their chronic disease.

**TABLE 1:** Demographic information, type of facemasks used, and exemption from wearing facemasks in total study population of people with primary ciliary dyskinesia (n = 282) and divided in adults (aged 18 years or older) and children (aged 17 years or younger). COVID-19 and Primary Ciliary Dyskinesia Study, Switzerland, 2020-2021.

	Total N = 282	Adults (≥18 years) N = 210	Children (<18 years) N = 72
Age, median (IQR)	32 (17–38)	40 (29–51)	11 (7–14)
Sex, female	179 (63)	145 (69)	34 (47)
Country of residence			
United Kingdom	62 (22)	51 (24)	11 (15)
Germany	57 (20)	30 (14)	27 (38)
United States	45 (16)	31 (15)	14 (19)
Switzerland	21 (7)	16 (8)	5 (7)
Australia	12 (4)	9 (4)	3 (4)
Italy	9 (3)	6 (3)	3 (4)
Other European countries	58 (21)	51 (24)	7 (10)
Other non-European countries	18 (6)	16 (8)	2 (3)
Type of facemasks used (multiple ticks possible)			
Single-use mask certified (surgical mask)	128 (45)	93 (44)	35 (49)
Single-use mask non-certified	56 (20)	42 (20)	14 (19)
Filtering face piece (FFP or FFP2/FFP3)	57 (20)	46 (22)	11 (15)
Fabric mask with exchangeable filters	64 (23)	44 (21)	20 (28)
Fabric mask without exchangeable filters	136 (48)	98 (47)	38 (53)
Other (e.g. scarf or other clothes pieces)	7 (2)	3 (1)	4 (6)
Exempt from wearing a mask because of PCD (n = 279)			
No	178 (64)	124 (60)	54 (76)
Yes, personal exemption prescribed	2 (1)	1 (0)	1 (1)
Yes, all people with PCD in my region are exempt	32 (11)	25 (12)	7 (10)
Don’t know	67 (24)	58 (28)	9 (13)

Abbreviations; IQR: inter quartile range.

**TABLE 2:** Number of study participants from different countries, number of people reporting that people with primary ciliary dyskinesia are exempt from wearing facemasks in their region, and facemask requirements (status of October 30, 2020) and date of full country requirement in countries represented by study the participants. COVID-19 and Primary Ciliary Dyskinesia Study, Switzerland, 2020-2021.

Country	No. of study participants (total N = 282)	People with PCD are exempt from wearing mask^a^	Mask required?^b^	Date of full country requirement (yyyy-mm-dd)^b^
Australia	12	3	Parts of country	—
Austria	3	0	Full country	2020-03-30
Belgium	2	0	Full country	2020-05-06
Brazil	2	0	Parts of country	—
Canada	10	1	Parts of country	—
Cyprus	2	0	Parts of country	—
Denmark	6	3	Full country	2020-08-20
France	3	0	Full country	2020-05-11
Georgia	1	0	Full country	2020-04-20
Germany	57	0	Full country	2020-04-27
Greece	1	0	Full country	2020-04-27
Hungary	1	0	No, but recommended	Mandatory from 2020-11-11
Hong Kong	1	0	No, but recommended	
Ireland	4	1	Full country	2020-07-16
Israel	3	0	Full country	2020-04-01
Italy	9	1	Full country	2020-05-04
Netherlands	12	1	Full country	2020-06-01
Norway	6	1	No	
Poland	4	1	Full country	2020-04-16
Portugal	1	0	Full country	2020-05-04
South Africa	1	0	Full country	2020-05-01
Spain	4	0	Full country	2020-05-02
Sweden	5	1	No	
Switzerland	21	0	Full country	2020-04-03
Turkey	1	0	Full country	2020-04-03
United Kingdom	65	15	Full country	2020-06-15
United States	45	2	Parts of country	—

a Reported by participants in study questionnaire.

b Data source: https://masks4all.co/what-countries-require-masks-in-public/

### Facemask Usage

**FIGURE 1:**
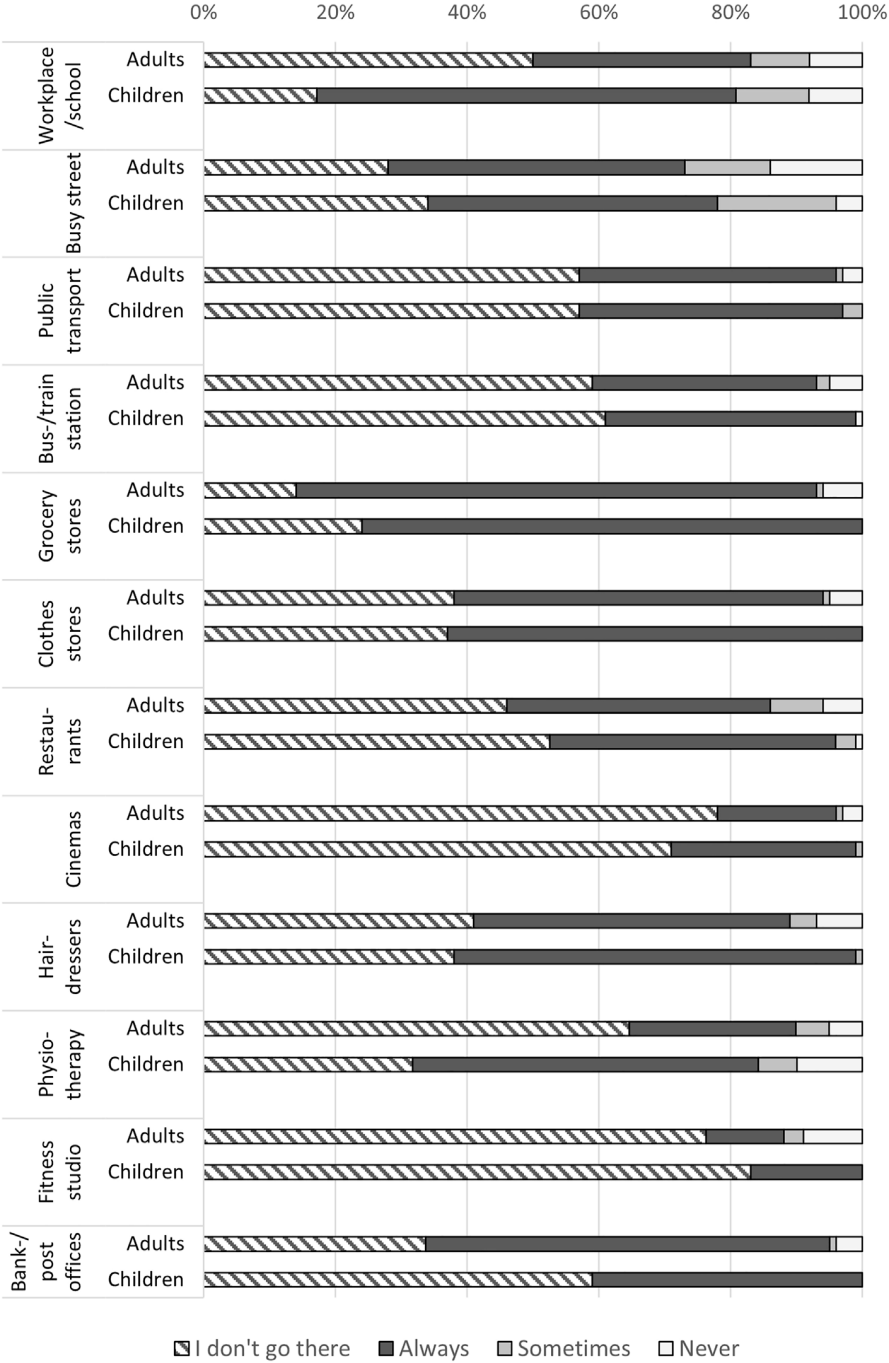
Frequency of people with primary ciliary dyskinesia wearing a facemask at different places among adults aged 18 or older and among children and adolescents aged 17 years or below (self-reported, October 2020; N = 282). COVID-19 and Primary Ciliary Dyskinesia Study, Switzerland, 2020-2021.

Almost all participants wore a facemask whenever they left their house, but they also avoided many places with a high risk of transmission (Figure 1). Taking public transport as example; 159 (57%) reported that they never used public transport, 108 (39%) reported that they always wore facemasks in public transport, 4 (1%) reported sometimes, and 7 (3%) reported never. The place visited by most participants was grocery stores where only 46 (16%) reported not going ever, 220 (79%) reported to always wear a facemask, and 12 (4%) reported to never wear a facemask. The place that was visited least often was fitness studios where 191 (78%) reported not to go ever, 31 (13%) reported always wearing a facemask, and 18 (7%) reported never wearing a facemask (Figure 1). There was little difference between children and adults except that more children went to school and physiotherapy than adults, while adults more often went to the bank or post office.

### Beliefs About Facemask Usage and Problems Reported Related to Their Use

Most participants supported the view that facemasks are effective in reducing transmission of SARS-CoV-2 with 227 (81%) agreeing that masks protect the person who wears the facemask, and 257 (91%) agreeing that masks protect others from getting infected (Table 3). Several reasons made it uncomfortable for people with PCD to wear a facemask. A third of participants (n = 94) reported that it was uncomfortable to wear a facemask because of their constantly runny nose, 88 (31%) because of cough, 61 (22%) because of difficulty in breathing, 7 (2%) because of headache, and 34 (12%) due to other problems including concentration problem, glasses fogging, pain or pressure from the mask, or sweating. 116 (40%) reported that their cough worsened when they wore a facemask for a long period of time. Consequently, 197 (70%) reported sometimes taking off their mask to blow their nose or to cough. One in four (65 people) found communication difficult with people who wear a facemask. This was particularly pronounced among participants with hearing impairment due to PCD (46%). Most participants paid for facemasks themselves, 49 (17%) received facemasks from their workplace or from medical staff, and 6 (2%) made their facemasks themselves. Very few (4%) reported that they cannot afford as many masks as needed, but 51 (22%) reported that the cost of facemasks was burdening their budget.

**TABLE 3:** Beliefs about facemask effectiveness and problems related to wearing a facemask reported by people with primary ciliary dyskinesia (N = 282). COVID-19 and Primary Ciliary Dyskinesia Study, Switzerland, 2020-2021.

	Total n (%)
I disagree that	
-facemasks protect me from getting infected with COVID-19	27 (10)
-facemasks protect me from spreading COVID-19 to others in case I am sick	11 (4)
I agree that	
-when I wear a facemask for a long time, my cough worsens	116 (41)
I find it uncomfortable to wear a facemask because of the following	
Runny nose	94 (33)
Cough	88 (31)
Difficulty breathing	61 (22)
Headache	7 (2)
Other problems^a^	34 (12)
Any of the above (runny nose, cough, difficulty breathing, headache, other)	137 (50)
I must sometimes take my facemask off because of runny nose or cough	197 (70)
How frequently do I take my facemask off because of runny nose or cough? (n = 195)	
Every few minutes	5 (3)
A few times per hour	75 (38)
Rarely, at long intervals	115 (59)
I have problems communicating with others when they wear a facemask	
Among all participants	65 (23)
Among participants with hearing impairment (n = 114)	52 (46)
Among participants without hearing impairment (n = 168)	13 (8)
Sometimes I am the only person who wears a facemask in places where it is not mandatory	118 (42)
Who pays for my facemasks (n = 251)	
They are provided or reimbursed	21 (8)
I pay them from my own pocket	193 (78)
I pay them but I also get some provided or reimbursed	28 (11)
I make them myself	6 (2)
Can I afford my facemasks (n = 238)	
I cannot afford as many as I need	8 (4)
Masks are heavy on my budget	51 (22)
Masks fit in my budget	176 (75)

a Other problems included difficulty concentrating (n = 2), sinus pain due to pressure from mask (n = 2), glasses fogging (n = 18), sweating (n = 2), fitting problems due to hearing aids or problems with size (n = 8), itchiness (n = 1), fear of stigmatizing (n = 1).

### Factors Associated With Facemask Usage

The only factor that was associated with not wearing a facemask in public was national facemask requirements (Table 4). In total, 17 (6%) of the participants reported that they never wore a facemask in two or more public places and 12 of these were from countries where facemasks were not required anywhere or were required only in certain regions. Demographic factors, beliefs about effectiveness, problems related to facemask wearing, self-reported lung function, or affordability were not associated with never wearing a facemask in public. Even participants who reported that people with PCD in their region were exempt from wearing a facemask mostly reported to always wear a mask (91%). Of the two people who reported having a personal facemask exemption, one reported sometimes wearing a facemask, and the other reported never wearing a facemask.

**TABLE 4:** Factors associated with not wearing a facemask in public among people with primary ciliary dyskinesia (N = 282). COVID-19 and Primary Ciliary Dyskinesia Study, Switzerland, 2020-2021.

	Wore a facemask in public^a^ N = 252	Never wore a facemask in one public place N = 13	Never wore a facemask in two or more public places N = 17	*p*-value^b^
Age group				0.360
0–17 years	64 (89)	6 (8)	2 (3)	
18–49 years	132 (89)	5 (3)	11 (7)	
Above 49 years	56 (90)	2 (3)	4 (6)	
Sex				0.132
Male	87 (85)	8 (8)	7 (7)	
Female	164 (92)	5 (3)	10 (6)	
Exempt from wearing a facemask because of PCD				0.004
No	161 (90)	10 (6)	7 (4)	
Yes, personal exemption	0	1 (50)	1 (50)	
Yes, people with PCD in my region are exempt	29 (91)	1 (3)	2 (6)	
I don’t know	61 (91)	0	6 (9)	
Beliefs about wearing a facemask (disagree or strongly disagree)				
Facemasks protect me from getting infected with COVID-19	23 (85)	1 (4)	3 (11)	0.395
Facemasks protect me from spreading COVID-19 to others	8 (73)	1 (10)	2 (18)	0.100
When I wear a facemask, my cough worsens	104 (90)	5 (4)	7 (6)	1.000
Uncomfortable to wear a facemask because of cough, runny nose, or other reason (n = 274)				0.780
No	124 (91)	7 (5)	6 (4)	
Yes	124 (91)	5 (4)	8 (6)	
Self-reported FEV_1_ at study entry (n = 192)				1.000
FEV_1_ ^a^ above 40% predicted	151 (87)	11 (6)	12 (7)	
FEV_1_ ^a^ below 40% predicted	16 (89)	1 (6)	1 (6)	
Difficult to communicate with others when they wear a facemask				0.214
No	194 (89)	12 (6)	11 (5)	
Yes	58 (89)	1 (2)	6 (9)	
Affordability of facemasks				0.727
I cannot afford as many as I need	9 (100)	0	0	
Facemasks are heavy on my budget	44 (86)	4 (8)	3 (6)	
Facemasks fit in my budget	163 (92)	8 (4)	7 (4)	
Country specific facemask recommendation				<0.001
Facemasks required in whole country	227 (93)	12 (5)	5 (2)	
Facemasks required in parts of country	20 (77)	0	6 (23)	
Facemasks not required	5 (42)	1 (8)	6 (50)	

^a^Busy street, public transport, Bus-/train station grocery store, clothes store, restaurants, cinemas, hairdressers, physiotherapists, fitness studios, bank-/post offices.

b Fischer’s exact if cell-counts were less than 10.

a FEV_1_: forced exhaled volume in one second

## Discussion

This international participatory study found that people with PCD carefully protected themselves against COVID-19 by avoiding many places and wearing facemasks in public. Many participants experienced problems with wearing a facemask due to their chronic recurrent cough, runny nose, and difficulty breathing but this did not prevent them from wearing a facemask. Facemask usage was not associated with age, sex, disease severity, or negative beliefs about facemask effectiveness. The only factor associated with facemask usage in public was living in a country with no national facemask requirement.

### Comparison With Other Studies and Interpretation of Results

To our knowledge, this is the first study to describe facemask usage and related problems in people with a chronic respiratory disease during the COVID-19 pandemic using original data. In a US survey of 1,056 adults from the general population conducted in May 2020, 825 (79%) reported to wear a facemask in public. The study included no information on places where facemasks were worn or frequency of use [36] which makes it difficult to compare with results from our study that showed that facemask usage differed by type of public place. In a cross-sectional online survey from Brazil conducted in July 2020 also in the general population, 1,266 of 1,277 (99%) said that they used facemasks but with no specification of where and how often [12]. The study further showed that two thirds of the participants (67%) were bothered by facemask in some way; 55% because of shortness of breath, 50% because of pain around the ears, and 44% because of glasses fogging. In our study, only 50% of people with PCD reported that facemasks were uncomfortable for any reason. A possible explanation may be that people with PCD are used to breathing problems in general and their threshold for what is uncomfortable may be higher. We believe it could also be, that they are very conscious of the gains related to facemask use and thus more tolerant towards discomfort.

Studies show that risk perception, disbelief in facemask effectiveness, and presence of national facemask policies are associated with adherence to wearing facemasks [37, 38]. People with PCD were considered at high risk of severe COVID-19 [27] and most of the participants in our study supported the opinion that facemasks are effective in preventing transmission of SARS-CoV-2. This may explain why almost all wore a facemask in public even if many reported problems because of chronic cough and rhinitis. Severity of lung problems could also have caused people with PCD to be less likely to wear a facemask which has been seen in relation to chronic obstructive pulmonary disease [16], however we found no evidence that people with FEV1 below 40% predicted were less likely to wear a facemask in public. The only factor that was associated with not wearing a facemask in our study was lack of public policies on facemask requirement. The same was found in other studies comparing facemask use in different countries [39–41]. Although the World Health Organisation recommends the use of facemasks in public [9], people with PCD, as well as people from the general population, might be more likely to follow national guidelines. This may be a reason for the difference in frequency of facemask usage between countries with and without national facemask requirements.

During the pandemic, discussion has evolved around facemasks exceptions for people with chronic lung diseases, which could have influenced the frequency of facemask use in people with PCD [42]. However, only two participants in our study had a personal medical exemption from wearing a facemask, and only one of them never wore a facemask in public. One in ten reported that people with PCD were exempt from wearing a face mask in their region, but most of these (91%) always wore a facemask in public despite this exemption. Among this group of people with a rare disease, of which many experienced breathing problems while wearing a mask, few exerted the right to not wear a facemask in public.

### Strengths and Limitations

A major strength of this study is the large sample size of people with a rare respiratory disease from different countries. It is difficult to recruit people with rare diseases for research, but the COVID-PCD is a participatory project that was initiated, designed, and tested in collaboration with people who have PCD. This boosted the study participation. Another strength is that the study is translated into five different languages (English, German, Spanish, Italian, and French). Our questionnaire included detailed questions about facemask use that made it possible to comprehensively understand facemask usage and related problems in a high-risk population. One limitation of the study is that our results are based on self-reported data, not real-life observations, but there was little risk of recall bias as the survey was sent out during a period when facemask use was compulsory in most countries and participants were wearing facemasks every day (October 2020). The study was anonymous so the risk of social desirability bias where answers would be influenced on what is socially accepted (e.g. all should wear facemasks) was low. Another limitation of our study was the response rate of 53% which may have led to selection bias.

### Conclusion

The findings from this international study suggest that people with a chronic respiratory disease carefully shield themselves against COVID-19 by avoiding public places and wearing facemasks. Participants who did not wear facemasks mainly came from countries without a national facemask requirement. National policies mandating facemask use in public are important for universal use to protect high-risk populations from SARS-CoV-2 infections.

## COVID-PCD Patient Advisory Group (in Alphabetical Order):

Sara Bellu, Associazione italiana Discinesia Ciliare Primaria Sindrome di Kartagener Onlus Italy; Fiona Copeland, PCD Family Support Group UK, UK; Katie Dexter, PCD Family Support Group UK; Lucy Dixon, PCD Family Support Group UK; Susanne Grieder, Selbsthilfegruppe Primäre Ciliäre Dyskinesie, Switzerland; Catherine Kruljac, PCD Australia Primary Ciliary Dyskinesia, Australia; Michelle Manion, PCD Foundation, USA; Bernhard Rindlisbacher, Selbsthilfegruppe Primäre Ciliäre Dyskinesie, Switzerland; Hansruedi Silberschmidt, Verein Kartagener Syndrom und Primäre Ciliäre Dyskinesie, Germany.

## Abbreviations

IQR, inter quartile range; COVID-19, coronavirus disease 2019; PCD, primary ciliary dyskinesia; SD, standard deviation

## References

[1] Chu DK Akl EA Duda S Solo K Yaacoub S Schünemann HJ Physical Distancing, Face Masks, and Eye protection to Prevent Person-To-Person Transmission of SARS-CoV-2 and COVID-19: a Systematic Review and Meta-Analysis. The Lancet (2020). 395(10242):1973–87. 10.1016/s0140-6736(20)31142-9 PMC7263814 32497510

[2] Leung NHL Chu DKW Shiu EYC Chan K-H McDevitt JJ Hau BJP Respiratory Virus Shedding in Exhaled Breath and Efficacy of Face Masks. Nat Med (2020). 26(5):676–80. 10.1038/s41591-020-0843-2 32371934 PMC8238571

[3] Chaabna K Doraiswamy S Mamtani R Cheema S. Facemask Use in Community Settings to Prevent Respiratory Infection Transmission: A Rapid Review and Meta-Analysis. Int J Infect Dis (2020). 104:198–206. 10.1016/j.ijid.2020.09.1434 32987183 PMC7518963

[4] Howard J Huang A Li Z Tufekci Z Zdimal V van der Westhuizen HM An Evidence Review of Face Masks against COVID-19. Proc Natl Acad Sci U S A (2021). 118(4). 10.1073/pnas.2014564118 PMC7848583 33431650

[5] Leffler CT Ing E Lykins JD Hogan MC McKeown CA Grzybowski A. Association of Country-wide Coronavirus Mortality with Demographics, Testing, Lockdowns, and Public Wearing of Masks. Am J Trop Med Hyg (2020). 103(6):2400–11. 10.4269/ajtmh.20-1015 33124541 PMC7695060

[6] Rader B White LF Burns MR Chen J Brilliant J Cohen J Mask-wearing and Control of SARS-CoV-2 Transmission in the USA: a Cross-Sectional Study. The Lancet Digital health (2021). 10.1016/s2589-7500(20)30293-4 PMC7817421 33483277

[7] Masks4all. What Countries Require Masks in Public or Recommend Masks? [Webpage] (2021). [updated 17-02-2021; cited 17-02-2021 17-02-2021]. Available from: https://masks4all.co/what-countries-require-masks-in-public/ (Accessed February 17, 2021).

[8] Chen N Zhou M Dong X Qu J Gong F Han Y Epidemiological and Clinical Characteristics of 99 Cases of 2019 Novel Coronavirus Pneumonia in Wuhan, China: a Descriptive Study. The Lancet (2020). 395(10223):507–13. 10.1016/s0140-6736(20)30211-7 PMC7135076 32007143

[9] WHO. Mask Use in the Context of COVID-19. Geneva (2020). Contract No.: WHO/2019-nCoV/IPC_Masks/2020.5.

[10] Zhou F Yu T Du R Fan G Liu Y Liu Z Clinical Course and Risk Factors for Mortality of Adult Inpatients with COVID-19 in Wuhan, China: A Retrospective Cohort Study. London, England: Lancet (2020). 10.1016/S0140-6736(20)30566-3 PMC7270627 32171076

[11] Aveyard P Gao M Lindson N Hartmann-Boyce J Watkinson P Young D Association between Pre-existing Respiratory Disease and its Treatment, and Severe COVID-19: a Population Cohort Study. Lancet Respir Med (2021). 9(8):909–23. 10.1016/s2213-2600(21)00095-3 33812494 PMC8016404

[12] Cotrin P Bahls AC da Silva Dd. O Girão VMP Pinzan-Vercelino CRM de Oliveira RCG The Use of Facemasks during the COVID-19 Pandemic by the Brazilian Population. J Multidiscip Healthc (2020). 13:1169–78. 10.2147/jmdh.s281524 33116562 PMC7585273

[13] Chaiyabutr C Sukakul T Pruksaeakanan C Thumrongtharadol J Boonchai W. Adverse Skin Reactions Following Different Types of Mask Usage during the COVID-19 Pandemic. J Eur Acad Dermatol Venereol (2020). 35:e176. 10.1111/jdv.17039 33220083 PMC7753376

[14] Bequignon E Dupuy L Zerah-Lancner F Bassinet L Honoré I Legendre M Critical Evaluation of Sinonasal Disease in 64 Adults with Primary Ciliary Dyskinesia. J Clin Med (2019). 8(5). 10.3390/jcm8050619 PMC6571605 31067752

[15] Kiedrowski MR Bomberger JM. Viral-Bacterial Co-infections in the Cystic Fibrosis Respiratory Tract. Front Immunol (2018). 9:3067. 10.3389/fimmu.2018.03067 30619379 PMC6306490

[16] Malay DS. Why My Patient Chose Not Wear a Face Mask. J Foot Ankle Surg (2021). 60(4):649. 10.1053/j.jfas.2021.05.008 34217436 PMC8268318

[17] Goutaki M Meier AB Halbeisen FS Lucas JS Dell SD Maurer E Clinical Manifestations in Primary Ciliary Dyskinesia: Systematic Review and Meta-Analysis. Eur Respir J (2016). 48(4):1081–95. 10.1183/13993003.00736-2016 27492829

[18] Kuehni CE Frischer T Strippoli M-PF Maurer E Bush A Nielsen KG Factors Influencing Age at Diagnosis of Primary Ciliary Dyskinesia in European Children. Eur Respir J (2010). 36(6):1248–58. 10.1183/09031936.00001010 20530032

[19] Lucas JS Walker WT Kuehni CE Lazor R. In: Cordier J-F, editor. Primary Ciliary Dyskinesia. Lausanne, Switzerland: European Respiratory Society (2011). p. 373. 2011-12-01 00:00:00.

[20] Rubbo B Best S Hirst RA Shoemark A Goggin P Carr SB Clinical Features and Management of Children with Primary Ciliary Dyskinesia in England. Arch Dis Child (2020). 105(8):724–9. 10.1136/archdischild-2019-317687 32156696

[21] Shapiro AJ Davis SD Ferkol T Dell SD Rosenfeld M Olivier KN Laterality Defects Other Than Situs Inversus Totalis in Primary Ciliary Dyskinesia. Chest (2014). 146(5):1176–86. 10.1378/chest.13-1704 24577564 PMC4219335

[22] Boon M Smits A Cuppens H Jaspers M Proesmans M Dupont LJ Primary Ciliary Dyskinesia: Critical Evaluation of Clinical Symptoms and Diagnosis in Patients with normal and Abnormal Ultrastructure. Orphanet J rare Dis (2014). 9:11. 10.1186/1750-1172-9-11 24450482 PMC4016480

[23] Davis SD Rosenfeld M Lee H-S Ferkol TW Sagel SD Dell SD Primary Ciliary Dyskinesia: Longitudinal Study of Lung Disease by Ultrastructure Defect and Genotype. Am J Respir Crit Care Med (2019). 199(2):190–8. 10.1164/rccm.201803-0548oc 30067075 PMC6353004

[24] Halbeisen FS Goutaki M Spycher BD Amirav I Behan L Boon M Lung Function in Patients with Primary Ciliary Dyskinesia: an iPCD Cohort Study. Eur Respir J (2018). 52(2). 10.1183/13993003.01040-2018 30049738

[25] Kouis P Goutaki M Goutaki M Halbeisen FS Gioti I Middleton N Prevalence and Course of Disease after Lung Resection in Primary Ciliary Dyskinesia: a Cohort & Nested Case-Control Study. Respir Res (2019). 20(1):212. 10.1186/s12931-019-1183-y 31533829 PMC6751891

[26] Pifferi M Bush A Mariani F Piras M Michelucci A Cangiotti A Lung Function Longitudinal Study by Phenotype and Genotype in Primary Ciliary Dyskinesia. Chest (2020). 158(1):117–20. 10.1016/j.chest.2020.02.001 32059959

[27] ELF. COVID-19 and Lung Disease Q&A UK: European Lung FOundation (2021). [updated 10/03/2021; cited 2021 14-09-2021]. Available from: https://europeanlung.org/en/information-hub/covid-19/expert-information/covid-19-and-lung-disease-qa/ (Accessed September 14, 2021).

[28] Bachtiger P Adamson A Maclean WA Kelshiker MA Quint JK Peters NS. Determinants of Shielding Behaviour during the COVID-19 Pandemic and Associations with Wellbeing in >7,000 NHS Patients: 17-week Longitudinal Observational Study. JMIR Public Health Surveill (2021). 7:e30460. 10.2196/30460 34298499 PMC8454693

[29] Pedersen ESL Collaud ENR Mozun R Ardura-Garcia C Lam YT Harris A COVID-PCD: a Participatory Research Study on the Impact of COVID-19 in People with Primary Ciliary Dyskinesia. ERJ Open Res (2021). 7(1). 10.1183/23120541.00843-2020 PMC7983255 33778058

[30] Pedersen ESL Goutaki M Harris AL Dixon L Manion M Rindlisbacher B SARS-CoV-2 Infections in People with PCD: Neither Frequent, Nor Particularly Severe. Eur Respir J (2021). 58:2004548. 10.1183/13993003.04548-2020 33833032 PMC8034057

[31] Riccio MP Borrelli M Fioretti MT Del Bene M Bravaccio C Poeta M Is Quarantine for COVID-19 Pandemic Associated with Psychological Burden in Primary Ciliary Dyskinesia? Int J Environ Res Public Health (2020). 17(21). 10.3390/ijerph17218099 PMC7663033 33153080

[32] von Elm E Altman DG Egger M Pocock SJ Gøtzsche PC Vandenbroucke JP. The Strengthening the Reporting of Observational Studies in Epidemiology (STROBE) Statement: Guidelines for Reporting Observational Studies. J Clin Epidemiol (2008). 61(4):344–9. 10.1016/j.jclinepi.2007.11.008 18313558

[33] Harris PA Taylor R Thielke R Payne J Gonzalez N Conde JG. Research Electronic Data Capture (REDCap)-A Metadata-Driven Methodology and Workflow Process for Providing Translational Research Informatics Support. J Biomed Inform (2009). 42(2):377–81. 10.1016/j.jbi.2008.08.010 18929686 PMC2700030

[34] Routledge R. Fisher's Exact Test. Encyclopedia of Biostatistics (2005).

[35] StataCorp. In: College Station TSL, editor. Stata Statistical Software: Release 15. College Station, TX: StataCorp LLC (2017).

[36] Latkin CA Dayton L Yi G Colon B Kong X. Mask Usage, Social Distancing, Racial, and Gender Correlates of COVID-19 Vaccine Intentions Among Adults in the US. PloS one (2021). 16(2):e0246970. 10.1371/journal.pone.0246970 33592035 PMC7886161

[37] Barceló J Sheen GC-H. Voluntary Adoption of Social Welfare-Enhancing Behavior: Mask-Wearing in Spain during the COVID-19 Outbreak. PloS one (2020). 15(12):e0242764. 10.1371/journal.pone.0242764 33259531 PMC7707551

[38] Hornik R Kikut A Jesch E Woko C Siegel L Kim K. Association of COVID-19 Misinformation with Face Mask Wearing and Social Distancing in a Nationally Representative US Sample. Health Commun (2021). 36(1):6–14. 10.1080/10410236.2020.1847437 33225745

[39] MacIntyre CR Nguyen P-Y Chughtai AA Trent M Gerber B Steinhofel K Mask Use, Risk-Mitigation Behaviours and Pandemic Fatigue during the COVID-19 Pandemic in Five Cities in Australia, the UK and USA: A Cross-Sectional Survey. Int J Infect Dis (2021). 106:199–207. 10.1016/j.ijid.2021.03.056 33771668 PMC7985682

[40] Annaka S. Public Awareness of Mask Usage in 29 Countries. medRxiv (2021). 10.1101/2021.03.06.21253037

[41] Covid-19: Global Behaviours Around Face Mask Use. London: Imperial College London; (2020).

[42] Guzman-Cottrill JA Malani AN Weber DJ Babcock H Haessler SD Hayden MK Local, State and Federal Face Mask Mandates during the COVID-19 Pandemic. Infect Control Hosp Epidemiol (2021). 42(4):455–6. 10.1017/ice.2020.1403 33397525 PMC7844166

