# Supplementary material for: Facemask Usage Among People With Primary Ciliary Dyskinesia During the COVID-19 Pandemic: A Participatory Project
Source: Int J Public Health. 2021 Dec 15;66:1604277. doi: 10.3389/ijph.2021.1604277 (PMC8715719; doi:10.3389/ijph.2021.1604277)
Supplement: Supplementary file 1 [file DataSheet1.docx]

**Supplementary file 1**: Formulation of questions and answers from the special questionnaire on facemask use sent to participants in October 2020.

| **Topic** | **Question formulation for adults aged 18 years and above and adolescents aged 14-17 years.** | **Question formulation for children (completed by parents)** | **Answer categories** |
| --- | --- | --- | --- |
| Date | Date of completing the questionnaire |  | Date |
| Facemask wearing | - | *Does your child sometimes wear a face mask? | 1)No, my child is not old enough to wear a face mask 2)Yes, my child sometimes wear a face mask |
| Exemption | Are you exempt from wearing a mask because you have primary ciliary dyskinesia? | Is your child exempt from wearing a mask in your region because he/she has Primary Ciliary Dyskinesia? | 1)No, I have to wear a mask where it is mandatory 2)Yes, I have a personal mask exemption prescribed by my doctor 3)Yes, people with PCD in my region can choose if they want to wear a mask 4)I don’t know |
| Place and frequency of facemask wearing | Where and how often do you wear a mask? Asked for the following public places: Grocery stores, Clothes shops, Restaurants, Bars, Public transport, Train stations, Cinemas, Hairdressers, Physiotherapy, Fitness centres, Yoga classes, Team sports, Contact sports, Bank-/post offices, My school, My workplace, Busy streets, Parks | Where and how often does your child wear a mask? Asked for the following public places: Grocery stores, Clothes shops, Restaurants, Bars, Public transport, Train stations, Cinemas, Hairdressers, Physiotherapy, Fitness centres, Yoga classes, Team sports, Contact sports, Bank-/post offices, My school, My workplace, Busy streets, Parks | 1)Always 2)Sometimes 3)Never 4)I don’t go there |
| Kind of facemask | What kind of mask do you wear? (tick all that apply) | What kind of mask does your child wear? (tick all that apply) | 1)Single-use mask certified 2)Single-use mask non-certified 3)Filtering face piece (FFP or FFP2/FFP3) 4)Fabric mask with exchangeable filters 5)Fabric mask without exchangeable filters 6)Other |
| Problems with facemasks | Do you find it uncomfortable to wear a mask because of your health problems? (tick all that apply) | Does your child find it uncomfortable to wear a mask because of health problems? (tick all that apply) | 1)No, I do not find it uncomfortable 2)Yes, because of my runny nose 3)Yes, because of my cough 4)Yes, because of other health problems |
| Problems with facemasks | Are there other reasons that make it uncomfortable for you to wear a mask | Are there other reasons that makes it uncomfortable for your child to wear a mask? | Free text |
| Conse- quence of facemask | What do you usually do when you have a runny nose or cough and wear a mask? | What does your child usually do when he/she has a runny nose or cough and wear a mask? | 1)When I have a runny nose or cough, I avoid wearing a mask 2)I sniff in and swallow the mucus or supress the cough and do not take the mask off 3)I sniff in and swallow the mucus or supress the cough but I also take the mask off from time to time to clear my nose 4)I take the mask off frequently to clear my nose |
| Take off facemasks | How often do you take the mask off to clear your nose or spit out mucus when you have cough or a runny nose? | How often does your child take the mask off to clear the nose or spit out mucus when he/she has cough or a runny nose? | 1)Every few minutes 2)A few times per hour 3)Rarely, a long intervals |
| Beliefs about facemasks | How much do you agree with the following sentences?  -Masks protect me from getting infected by COVID-19  -Masks protect me from spreading COVID-19 to others in case I am sick  -When I wear a mask for a long time, my cough worsens because I supress my cough or because I do not clear my nose | How much do you agree with the following sentences?  - Masks protect my child from getting infected by COVID-19  - Masks protect my child from spreading COVID-19 to others in case he/she is sick  - When my child wears a mask for a long time, his/her cough worsens because he/she suppresses the cough or because he/she does not clear the nose | 1)I strongly agree 2)I somewhat agree 3)I neither agree nor disagree 4)I somewhat disagree 5)I strongly disagree 6)I don’t know |
| Hearing | Do you have a hearing impairment? | Does your child have a hearing impairment? | 1)No 2)Yes |
| Commu- nication | Do you find communicating with other people more difficult when they wear a mask? | Does your child find communicating with other people more difficult when they wear a mask? | 1)No 2)Yes, but I can deal with this problem quite easily 3)Yes, this is difficult for me |
| Only one wearing facemask | Are you sometimes the only one wearing a mask when it is not mandatory? | Is your child sometimes the only one wearing a mask when it is not mandatory? | 1)No, other people also wear a mask in most places I go to 2) Yes, sometimes 3)Yes, often |
| Financing facemask | Do you have to pay for masks out of your own pocket? | Do you have to pay for masks for your child out of your own pocket? | 1)No, they are provided or I am reimbursed 2)Yes, I pay for them from my own pocket 3)Yes, mainly from my own pocket but some are also provided or I am reimbursed 4)Yes, but I make them myself |
| Affor-dability | Are masks affordable for you? | Are masks affordable for you? | 1)No, I cannot afford to buy as many masks as I need 2) Yes, but they are heavy on my budget 3) Yes, they fit in my budget |
| Comments | Please write any additional comments you may have about mask use | Please write any additional comments you may have about mask use or comments to this questionnaire | Free text |

The questions described in this table were adapted to three age-groups: children aged 0-14 years (completed by parents), adolescents aged 14-17 years, and adults aged 18 or above. *Only asked in the questionnaire for children aged 0-14 years

**Supplementary file 2**: Comparison of people who completed the facemask questionnaire and those who did not among participants included in the COVID-PCD study by October 10, 2020 (N=572).

|  | **Completed facemask questionnaire** | **Did not complete facemask questionnaire** | **P-value*** |
| --- | --- | --- | --- |
|  | N=282 | N=290 |  |
| **Age**, median (IQ range) | 32 (17-48) | 22 (8-37) | <0.001 |
|  |  |  |  |
| **Sex** |  |  | 0.363 |
| Female | 179 (51) | 172 (49) |  |
| Male | 102 (47) | 117 (53) |  |
|  |  |  |  |
| **Country of residence** |  |  | 0.004 |
| United Kingdom | 62 (45) | 77 (55) |  |
| Germany | 57 (70) | 25 (30) |  |
| USA | 45 (44) | 58 (56) |  |
| Switzerland | 21 (50) | 21 (50) |  |
| Australia | 12 (44) | 15 (56) |  |
| Italy | 9 (39) | 14 (61) |  |
| Other European countries | 58 (53) | 51 (47) |  |
| Other non-European countries | 18 (40) | 27 (60) |  |

This table shows number and row percent unless otherwise stated. Student’s t-test for age and chi squared test for sex and country of residence.
